# Translation and Adaptation of the Japanese Version of the Revised Parental Stressor Scale: Pediatric Intensive Care Unit

**DOI:** 10.7759/cureus.64389

**Published:** 2024-07-12

**Authors:** Mitsuki Ikeda, Haruhiko Hoshino, Gen Aikawa, Yujiro Matsuishi, Misaki Kotani, Yuki Enomoto, Nobutake Shimojo, Yoshiaki Inoue

**Affiliations:** 1 Department of Emergency and Critical Care Medicine, Faculty of Medicine, University of Tsukuba, Tsukuba, JPN; 2 Department of Nursing, University of Tsukuba Hospital, Tsukuba, JPN; 3 Adult Nursing/Acute Care, Department of Nursing, Faculty of Medical Technology, Teikyo University, Itabashi, JPN; 4 College of Nursing, Kanto Gakuin University, Yokohama, JPN; 5 Adult and Elderly Nursing, Faculty of Nursing, Tokyo University of Information Sciences, Chiba, JPN; 6 Laboratory of Artificial Intelligence, Intelligent and Mechanical Interaction Technologies (IMIS), University of Tsukuba, Tsukuba, JPN

**Keywords:** stressor, stress, critically ill children, post-intensive care syndrome-family, post-intensive care syndrome, pediatric intensive care unit

## Abstract

Introduction

The physical, cognitive, and psychiatric disorders that occur in patients after leaving the intensive care unit (ICU) are collectively called post-intensive care syndrome (PICS). Moreover, PICS-family (PICS-F) refers to the long-term psychological and social disorders that occur in the family. The symptoms of PICS-F can be psychological, and stress is a known cause of these symptoms. The Parental Stressor Scale: Pediatric Intensive Care Unit (PSS: PICU) was developed to assess stress levels and related factors among the families of patients admitted to the PICU. It has been translated into several languages and was revised in 2021. However, a Japanese version of the revised PSS: PICU (J-R-PSS: PICU) has not yet been developed. This study aimed to develop a J-R-PSS: PICU and to test its acceptability for clinical use.

Materials and methods

A back-translation method, involving initial translation, review by bilingual experts, and subsequent re-translation to ensure accuracy, was used to develop the J-R-PSS: PICU. Families with patients in the PICU for >48 hours between November and December 2021 and those who were transferred out of the ICU were recruited. Moreover, the study documents with a QR code for a web questionnaire were provided and explained to family members. Stress scores and stressors of family members were collected from web questionnaires using the PSS: PICU as the primary endpoint. Other information about the patients and their families was collected from clinical records and questionnaires. Participants and an expert panel evaluated the clarity of each item, and the expert panel evaluated the relevance of each item.

Results

Twenty family members who met the inclusion criteria and provided informed consent were included. The J-R-PSS: PICU was developed using a back-translation method. For clarity, all items were clarified after a single modification by an interdisciplinary team. For relevance, all the items had a content validity index at an item level of ≥0.8 and a scale level of 0.94. Alpha coefficients were 0.93 for the overall scale and 0.69-0.97 for its subscales.

Conclusion

We developed the J-R-PSS: PICU with high content validity and internal consistency using a back-translation method.

## Introduction

In recent years, physical, cognitive, and psychiatric disorders that occur in patients after leaving the intensive care unit (ICU) have been called post-intensive care syndrome (PICS), whereas long-term psychological and social disorders that occur among family members are called PICS-family (PICS-F) [1]. The symptoms of PICS include psychological symptoms such as acute stress disorder (ASD) and post-traumatic stress disorder (PTSD); stress is a known factor that causes these symptoms [1,2]. A previous study found that approximately 32% of families of patients admitted to the pediatric ICU (PICU) had ASD symptoms [3], 10-42% had PTSD symptoms [4-6], and families of patients admitted to the PICU were more likely to have PTSD symptoms than families of patients in general pediatric units (27% vs. 7%, p = 0.04 ) [7]. Admission to the PICU itself has been reported as a stressful experience for families [8,9].

Despite a lot of children and families suffering, there is not much of a scale to figure out the stress and stressor of families in PICU. The Parental Stressor Scale: Pediatric Intensive Care Unit (PSS: PICU) was developed in the United States in 1989 as a tool to assess stress levels and factors among the families of patients admitted to PICUs [10]. It has been translated into the languages of Malaysia, China, India, Spain, and other countries to figure out the stress and stressors of families [11-14], and a revised version was developed by Alzawad et al. in the United States in 2021 [15]. Using PSS: PICU revealed mothers and fathers in PICU experienced more stress symptoms than the parents in the general care unit each other [8,9]. It has been suggested that it may be important for both parents to receive preparation regarding the stresses and environmental changes they will undergo upon entering the PICU [8,9]. Understanding the stress factors of families in PICU at the beginning of admission may help prepare the strategies to prevent the risk of developing PTSD and discover the best time to intervene to help prevent PTSD after discharge [15]. However, a Japanese version of the PSS: PICU has not been developed, the stress felt by families of patients admitted to PICUs has not been quantitatively evaluated, and stress factors have not been clarified. This study aimed to develop a Japanese version of the PSS: PICU and to test its acceptability for clinical use.

## Materials and methods

Setting and participants

Research Design and Setting

The study design was a cross-sectional study. This study was conducted in the PICU of the University of Tsukuba Hospital, Tsukuba, Japan. The hospital has 800 beds, including eight PICU beds (six open beds and two private rooms). The PICU is a semi-closed system in which the intensive care management of critically ill pediatric patients is performed jointly by intensivists and other specialty doctors.

Participants

Families of patients admitted to the PICU for >48 hours between November and December 2021 and those who were transferred out of the ICU were prospectively recruited. We excluded families who did not visit during their patients’ stay in the PICU, those who could not read or write Japanese, those who had a patient who died, those discharged to the neonatal ICU, and those with a mental illness.

Sample Size

According to the guidelines, a sample size of 10-40 individuals is recommended for asking participants to rate the clarity of each item on a scale [16,17]. Moreover, to evaluate internal consistency by using Cronbach’s α for this scale when the number of items = 40 and Cronbach’s alpha at null hypothesis = 0, based on alpha <0.05 and power of at least 90.0%, a minimum sample size of 17 is sufficient to detect the expected value of Cronbach’s alpha = 0.70 [18]. Thus, convenience sampling was used for recruiting 20 parents. Moreover, 10 expert panels who are familiar with the contents and field regarding the scale and target population evaluated the clarity and relevance of each item [17].

Procedure

Instrument

The PSS: PICU consisted of six subscales and 39 items [15]. The six subscales were 1) child appearance, 2) sights and sounds, 3) procedures, 4) staff communication, 5) children’s behavior and emotional responses, and 6) parental role. Each item was rated on a six-point Likert scale ranging from 0 to 5 (0, not experienced; 1, not stressful; 2, minimally stressful; 3, moderately stressful; 4, very stressful; and 5, extremely stressful), with higher total scores indicating higher levels of stress.

Data Collection

At the time of the visit, more than 48 hours after admission to the PICU, the study documents were provided and explained to family members. A document containing a QR code for the encrypted and confidential web-based questionnaire was provided only after informed consent was obtained. If there was no contact within one week of providing the materials, a reminder phone call was made to the respondents to ascertain whether they had responded.

The stress scores and stressors of the family members were collected from web questionnaires using the PSS: PICU as the primary endpoint. Other information about patients and their families such as the Pediatric Risk of Mortality Ⅲ, which indicates the severity of patients, was collected from the clinical records and questionnaires.

Back Translation

**Figure 1:**
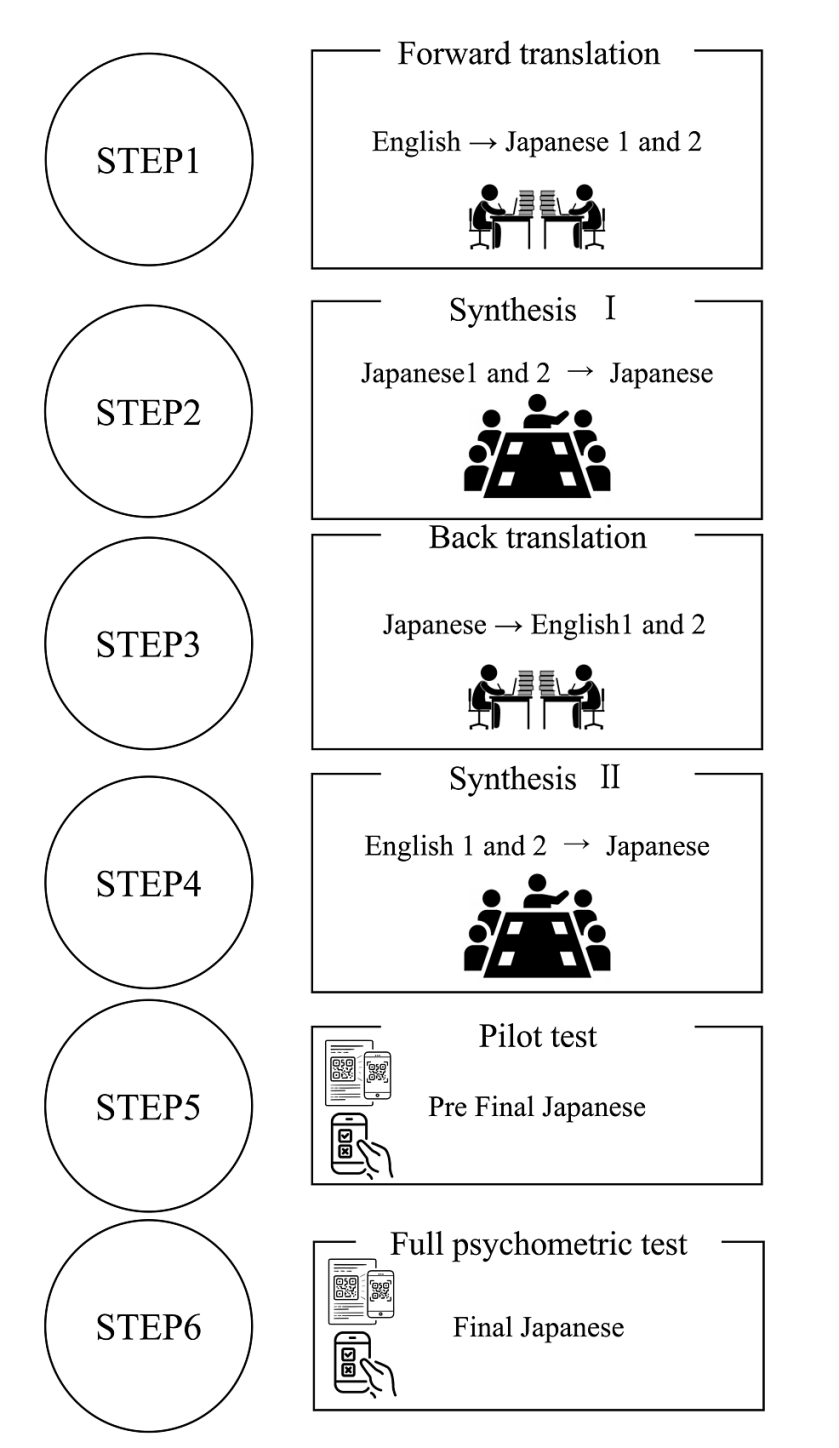
Back-translation method flow. Step 1 (forward translation): Two independent bilingual and bicultural translators whose mother language was the target language (TL/Japanese) forward-translated the original instrument from the source language (SL/English) to the TL. Step 2 (synthesis Ⅰ): Two translators from Step 1, a third bilingual and bicultural translator, and another research member compared the two forward-translated versions (TL1 and TL2) with the original version in the SL. The translation team discussed ambiguities and discrepancies in the words, sentences, and meanings until all problems were resolved. A preliminary translated version of the instrument in the TL (PI-TL) was generated using this process. Step 3 (back translation): Two other independent bilingual and bicultural translators, whose mother language was the SL, translated the PI-TL back into the SL. The translators were blinded to the original version. Two back-translated versions of the SL (B-TL1 and B-TL2) were produced using this process. Step 4 (synthesis Ⅱ): A multidisciplinary committee consisting of the members in steps 2 and 3, one TL monolingual member, and a developer of the original instrument compared B-TL1, B-TL2, and the original instrument. Steps 1–4 were repeated until no ambiguities or discrepancies were observed. A pre-final version of the instrument in the TL (P-FTL) was generated using this process. Step 5 (pilot test): To evaluate the instructions, items, and response format of the P-FTL for clarity, a pilot test was conducted with 20 individuals whose language was the TL and an expert panel familiar with the domains of the scale and the target group. Step 6 (full psychometric test): To revise and refine the items of the final version of the instrument in the TL (FTL), a full psychometric test of the P-FTL was conducted on the target population.

To develop the Japanese version of the revised PSS: PICU (J-R-PSS: PICU), a back-translation method was used according to the guidelines [17]. Throughout the back-translation procedure, the developed instrument was compared with the original versions in terms of wording, grammatical structure, semantic similarities, and biases. Discussions were conducted until ambiguities and inconsistencies were resolved by an interdisciplinary team (Figure 1).

Analysis plan

Validity: Content Validity (Face Validity)

The participants and an expert panel were asked to rate the clarity of each item on a scale of 1 to 4 (1, not at all; 2, somewhat; 3, fairly; and 4, exactly), with unclear points freely described. The expert panel consisted of one intensivist, two pediatricians, two nurse managers, and five PICU nurses. The results of the responses were calculated dichotomously, with 1 or 2 considered “unclear” and 3 or 4 considered “clear.” If an item was deemed unclear by more than 20% of the participants, it was revised by a translation team based on feedback. This process was repeated until it exceeded 80% for all items [17]. The expert panel comprised professionals (intensivists, pediatricians, and PICU nurses) familiar with the scale and target population domains.

Validity: Content Validity (Logical Validity)

The expert panel evaluated the relevance of each item (1, not relevant; 2, not assessable as relevant; 3, relevant but needing some modification; and 4, very relevant and concise). Those items rated 1 or 2 were modified. The content validity index (CVI) at the item (I-CVI) and scale (S-CVI) levels was also measured, and revisions were repeated until minimum acceptable indexes of 0.78 and 0.9 were achieved for the I-CVI and S-CVI, respectively [19].

Reliability

Cronbach’s α for all subscales and the whole scale were calculated to investigate internal consistency. Usually, an α-coefficient ≥0.7 is considered a reliable measure with internal consistency [20].

Statistical analyses were performed using IBM SPSS Statistics, Version 28.0 (released 2021, IBM Corp., Armonk, NY).

Ethical Considerations

The study protocol was approved by the Institutional Review Board of the University of Tsukuba Hospital (Approval No. R03-99). It conformed to the provisions of the Declaration of Helsinki.

## Results

Translation process

After receiving permission from the licensor of the original version [10] and the author of the revised version [15], the interdisciplinary team created a Japanese version using the back-translation method (Figure 1; see Appendix 1 and 2). In this study, we used only Steps 1-5, following the recommendation from the guidelines [17], to translate, adapt, and cross-validate the study instrument (Figure 1). An interdisciplinary team, including the original author, ensured that no clinical discrepancies existed in the final back-translated English version.

Participants

**Figure 2:**
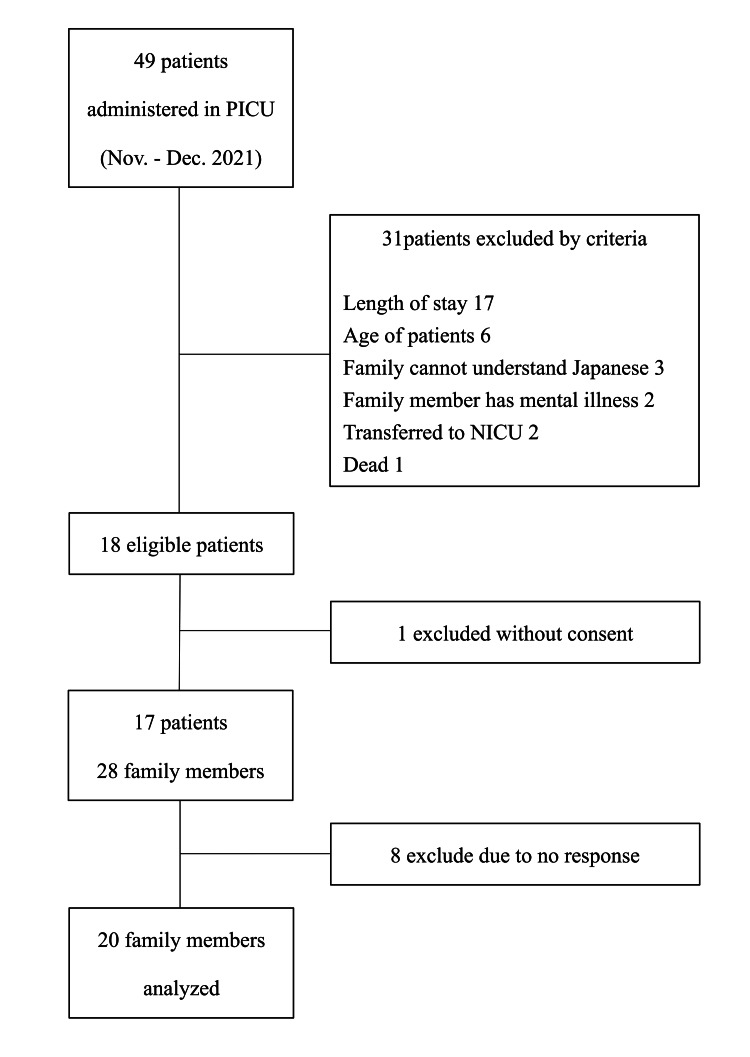
Inclusion flow. Dec.: December, NICU: neonatal intensive care unit, Nov.: November, PICU: pediatric intensive care unit

Of the 49 patients in the study, 17 met the eligibility criteria and 28 parents provided consent. The 20 family members who responded were included in the analysis. The response rate was 71% (Figure 2).

Characteristics of the Children

The median age of the children at enrollment (interquartile range (IQR)) was one year old (IQR, 0-6.5 years old). The most common diagnostic admission category was cardiac pathology (35%), and the most common cause of admission was scheduled surgery (54%). The median length of hospital stay was three days (IQR, 2.0-8.5 days). Approximately half of the patients were intubated (47%), 53% underwent surgery, 47% had a previous medical condition, and 41% had a history of hospitalization. Most patients had siblings (82%). The pediatric risk of mortality III median score was 12 (IQR, 7.0-18.5) (Table 1).

**Table 1:** Demographic characteristics of the participants. Values are presented as median (IQR) or n (%). IQR: interquartile range

Characteristics of the children and parents	Values
Children	(n = 17)
Age at enrollment (years, IQR)	1 (0–6.5)
Sex (%)	
Female	9 (53)
Male	8 (47)
Admission diagnostic category (%)	
Cardiological pathologies	6 (35)
Neurologic pathologies	4 (23)
Respiratory pathologies	3 (18)
Gastroenterological pathologies	2 (12)
Infection	1 (6)
Others	1 (6)
Admission route (%)	
Scheduled surgery	9 (54)
Emergency admission from outside the hospital	3 (18)
Pediatric general ward	3 (18)
Emergency surgery	1 (5)
Unscheduled admission from the operation room	1 (5)
Intubation (%)	8 (47)
Length of stay (day, IQR)	3 (2.0–8.5)
Surgical history (+, %)	9 (53)
Previous medical history (+, %)	8 (47)
Hospitalization history (+, %)	7 (41)
Having siblings (%)	14 (82)
Pediatric Risk of Mortality Ⅲ (IQR)	12 (7.0 – 18.5)
Parents	(n = 20)
Response time (minutes, IQR)	9.5 (7.3–27.8)
Age (years, IQR)	35 (32.3–36.0)
Gender (%)	
Mother	14 (70)
Father	6 (30)
Occupation (%)	
Housemaker	5 (25)
Part-time job	5 (25)
Company employee	4 (20)
Civil servant	2 (10)
Other	2 (10)
On maternity/paternity leave	1 (5)
Freelance	1 (5)
Annual income (US$ [ 140 Japanese Yen / 1 US$ ], %)	
〜21,000	11 (55)
21,000-35,000	3 (15)
35,000-70,000	3 (15)
70,000-100,000	1 (5)
100,000–140,000	1 (5)
Unanswered	1 (5)
Education (%)	
University	10 (50)
Graduate school	4 (20)
High School	2 (10)
Vocational school	2 (10)
Community college	1 (5)
Other	1 (5)

Characteristics of the Parents

Twenty parents, including 14 mothers (70%) and six fathers (30%), participated in the survey. The median response time was 9.5 minutes (IQR, 7.3-27.8 minutes), and the median parental age was 35 years old (IQR, 32.3-36.0 years old). Ten parents (50%) had graduated from university, and four (20%) had postgraduate degrees. Regarding their occupations, housewives (25%) and part-timers (25%) were the most common, and 55% of the respondents had an annual income of ≤3 million Japanese yen (Table 1).

Validity

Content Validity (Face Validity)

Regarding clarity, nine items were deemed unclear by >20% of the parents, and seven items were assessed as unclear by ≥20% of the expert panel. Half of the expert panels were male (50%), the median age of the expert panel at enrollment was 30 years old (IQR, 26-39 years old) and the median year of PICU experience was five years (IQR, 2-12 years). All the items were considered clear after a single modification by the interdisciplinary team. Furthermore, through discussions with an interdisciplinary team, we confirmed that the semantic content of the original and Japanese versions was equivalent.

Content Validity (Logical Validity)

Regarding logical validity, only one item ("20. Bruises on my child") had an I-CVI of 0.7. Based on the feedback from the expert panel, the items were revised by an interdisciplinary team. There were some comments such as “20. Bruises on my child," "21. Cuts on my child" and "22. Incisions on my child are difficult to differentiate in Japanese. Is it clinically acceptable for a child to have a blue bruise? Is the question item inappropriate in the first place?” Thus, the questions themselves were not changed a lot when presented in English, but the discussion took place in consultation between the multidisciplinary team in response to the comments, regarding the Japanese meaning and the way it was expressed. All the items then had an I-CVI of ≥0.8 and S-CVI of 0.94 (Table 2), so the J-R-PSS: PICU was confirmed to have high content validity.

**Table 2:** Validity and reliability of the Japanese version of the Revised Parental Stressor Scale: Pediatric Intensive Care Unit I-CVI: content validity index at item level, S-CVI: content validity index at scale level, SD: standard deviation

Types	Items		Values
Initial item set (n = 10, 40 items)	I-CVI	min	0.70
		max	1.00
		mean ± SD	0.93 ± 0.08
	S-CVI	―	0.93
Revised item set (n = 10, 40 items)	I-CVI	min	0.80
		max	1.00
		mean ± SD	0.94 ± 0.07
	S-CVI	―	0.94
Cronbach’s α (n = 20, 40 items)	Child appearance		0.91
	Sights and sounds		0.69
	Medical procedures		0.77
	Staff communication		0.97
	Child’s behavior and emotional responses		0.91
	Parental role		0.72
	Total		0.93

Reliability

Internal consistency was estimated using Cronbach’s α-coefficients, which were calculated for the entire scale and each of the six subscales. The α-coefficients were 0.93 for the overall scale and ranged between 0.69 and 0.97 for the subscales (Table 2). The α-coefficients for almost all subscales were ≥0.70, which indicates that the internal consistency of the J-R-PSS: PICU is reasonable (0.67-0.87) to high (0.73-0.95) [20].

Stressors of families in the PICU

Stressors of families in the PICU were also identified in the present study (Table 3). The scores for each questionnaire item were calculated using the mean and standard deviation of each subscale. The mean values for the subscales ranged between 0.97 and 3.13, and the wide range of values was due to the inclusion of zero (not experienced) responses. The parental role items were the most stressful factor for the participants in this study (3.13 ± 1.68), whereas they experienced less stress from staff communication (0.97 ± 1.70).

**Table 3:** The Japanese version of the Revised PSS: PICU subscale means and standard deviations SD: standard deviation

Subscale	Mean	SD
Child appearance	2.54	±2.09
Sights and sounds	1.85	±1.25
Medical procedures	2.14	±1.64
Staff communication	0.97	±1.7
Child’s behavior and emotional responses	2.51	±1.92
Parental role	3.13	±1.68

## Discussion

We developed the J-R-PSS: PICU using back translation based on the guidelines [17], taking into account linguistic and cultural differences and ensuring that there were no discrepancies in clinical use. Items that were unclear to the participants and expert panel were corrected by an interdisciplinary team (Table 2). The Cronbach’s α coefficients were also high, as in previous studies [10,13,14], and the internal consistency of the J-R-PSS: PICU was verified.

Regarding translation methods, the previous study used translation by people linguistically familiar with the source language, English [11,12], back translation [13,14], or asked bilingual parents to respond [13]. The present study was conducted using back-translation methods in line with the guidelines [17], and it can be said that linguistic and cultural differences were considered in the creation of the Japanese version.

In terms of validity, content validity (face validity) was modified by the multidisciplinary team and all items were clear for respondents and the expert panel. Moreover, the content validity (logical validity) score in this study was above the baseline I-CVI of 0.78 and S-CVI of 0.9 [19]. It was also comparable to the previous study, I-CVI which calculated from the answers of six nurses with extensive experience in pediatric neonatal nursing was 0.83 [14]. These showed that the content validity of the J-R-PSS: PICU was acceptable.

Concerning reliability, in the present study, the α-coefficients exceeded the criterion of 0.7 in the whole items and all subscales except “sights and sounds” of 0.69, which was reasonable to high [20]. In comparison with the previous study, Cronbach's α coefficients ranged from 0.83 to 0.99, with a total of 0.95 [10]; 0.75 to 0.93, with a total of 0.95 [11]; 0.47 to 0.94, with a total of 0.91 [13]; and 0.82 to 0.95, with a total of 0.94 [14], which were high despite the small sample size in this study.

The most commonly reported stressors for families with patients admitted to the PICU were medical procedures [9,21] and sights and sounds [8,13]. On the other hand, as in the original version [10], the role of parents was the highest in this study (Table 3), although the number of subjects was smaller. We believe that this is due to the inadequacy of the parental role, including limited contact with physical and psychological separation, and guilt toward children. In a recent study, families of patients in the PICU experienced mental and emotional overload, and the mental impact of being in the PICU was more burdensome than that of the physical environment [15]. However, it has been reported that this differs among facilities, countries, and regions and is attributed to environmental factors such as patient background and the therapeutic environment associated with the disease [10,13,14] and may also be influenced by sociocultural background. All of these factors call for more surveys in each region and facility.

A strength of this study is that it is the first in Japan to develop a scale that can quantitatively assess stress and identify stressors among families of patients in PICUs. Few studies have examined the mental status of the families of critically ill children in Japan. Families are an important part of a patient’s life, and the physical, emotional, and social stability of families as caregivers is essential for continued treatment and care of children. PICS-F symptoms persist from days after admission to years after discharge. Family PTSD is a risk factor with a bidirectional influence on PTSD development in pediatric patients [1]. Therefore, identifying and addressing the problems of families during admission will lead to the development and well-being of the patient’s physical and mental functions and those of the family. Thus, using the J-R-PSS: PICU to determine stress levels and stressors in the families of patients admitted to the PICU can identify the need for specific care and may contribute to the prevention or reduction of PICS-F, eventually leading to comfort in pediatric patients. Further clinical use of this scale and investigation of stress levels and stressors among families in the PICU are required. For example, quantitative stress score surveys allow healthcare providers to objectively determine trends in stressors experienced by the families of patients in the PICU and share information with the medical team. It could also clarify the impact of patient family stress during the PICU stay on post-discharge psychiatric symptoms and identify risk factors from subscale stressors to develop effective stress-relieving interventions. There is a need for studies that will lead to early care during admission to the PICU so that patients and their families do not suffer further from not only surgery and treatment but also PICS.

There are several limitations of this study. One of those is that only content validity and internal consistency were assessed. Therefore, it is necessary to conduct a full psychometric test using the back-translation method [17]. Further validity and reliability evaluations are required before clinical implementation and utilization. The current study was conducted with the sample size necessary to develop a scale calculated according to the guidelines [17]. For a small sample size, the age difference of the subjects is relatively small. The fact that the ratings were given by parents with a small age difference could conceivably influence the results. The median age of the children was one year old (IQR, 0-6.5 years old) and the parental age was 35 years old (IQR, 32.3-36.0 years old). Moreover, the mean age of the children was 4.5 years old, with a range of 0 to 14 years, and parental age was 35 years old, with a range of 24 to 46 years. Therefore, it is not significantly different from the previous study [11-14]. On the other hand, stress may be different for parents in older age groups or with a wide age range of respondents. If the sample size is increased in future surveys, family stressors may change. It is necessary to clarify the stress levels and factors in future surveys and to adjust and analyze the results based on the background information of patients and families in the PICU.

## Conclusions

In this study, we developed the J-R-PSS: PICU with high content validity and internal consistency through the back-translation method. Furthermore, this study offers valuable insights into quantitatively assessing stress and identifying stressors among families with patients admitted into the PICU. Using the J-R-PSS: PICU to determine the stress levels and stressors of families has led to the need for specific care and may contribute to the prevention or reduction of post-intensive care syndrome in families, eventually leading to the comfort of pediatric patients.

## Appendices

Appendix 1

**Table 4:** Back-translated Japanese version of the revised PSS: PICU Example responses: 1) For items that are “extremely stressful,” please circle 5. 2) For items that are “not stressful,” please circle 1. 3) For items that are “not experienced,” please circle 0. 0: not experienced, 1: not stressful, 2: minimally stressful, 3: moderately stressful, 4: very stressful, 5: extremely stressful

Subscale	Items
Child appearance	Q1. My child is not getting better
	Q2. The color of my child’s body appears different (pale, yellow, etc.)
	Q3. My child is very sick
	Q4. My child’s condition is getting worse
Sights and sounds	Q5. Seeing my child’s heart rate on the monitor
	Q6. The bright lights
	Q7. The uncomfortable bed
	Q8. Being unable to sleep
	Q9. The sudden sound of monitor alarms
	Q10. The limited privacy
	Q11. Seeing other parents in distress
	Q12. Seeing other sick children
	Q13. Frequent interruptions by staff entering and leaving the room
	Q14. My child’s admission the PICU was unexpected
Medical procedures	Q15. Tubes placed in my child (e.g., feeding tube, urinary catheter, IV)
	Q16. Suctioning
	Q17. Needles placed in my child for fluids, procedures, or tests
	Q18. Pounding on my child’s chest
	Q19. My child is connected to a machine to breath
	Q20. Bruises on my child
	Q21. Cuts on my child
	Q22. Incision wounds on my child
Staff communication	Q23. I feel that the doctors do not discuss my child’s illness with each other
	Q24. My child’s condition is not fully discussed with me
	Q25. I receive conflicting explanations about my child’s condition
	Q26. Not stating what is wrong with my child
	Q27. Not talking to me enough about my child’s treatment
Child’s behavior	Q28. My child appears confused
and emotional responses	Q29. My child is crying or whining
	Q30. My child is acting or looking as if in pain
	Q31. My child is restless
	Q32. My child is unable to talk
	Q33. My child is unable to cry
	Q34. My child appears scared
	Q35. My child appears sad
	Q36. My child appears to be depressed
Parental role	Q37. Not being able to take care of my child
	Q38. Not wanting to leave my child for even a moment
	Q39. Not being able to hold my child
	Q40. Not knowing the best way to help my child in their critical situation

Appendix 2

**Table 5:** 親のPICUストレス要因尺度 (日本語版PSS: PICU) 回答例 「最もストレスに感じる」質問項目は、5を選択してください 。 「ストレスに感じない」質問項目は、1 を選択してください。 「体験したことがない」質問項目は、0 を選択してください。 0 体験したことがない 1 ストレスに感じない 2 少しストレスに感じる 3 ややストレスに感じる 4 とてもストレスに感じる 5 最もストレスと感じる

下位尺度	質問項目
お子さまの様子	Q1. 子どもの具合が良くならない
	Q2. 子どもの身体の色が変わって見える(蒼白、真っ⻘、⻩色いなど)
	Q3. 子どもの具合が非常に悪い
	Q4. 子どもの健康状態が悪化している
ICUにおける周囲の様子と音	Q5. モニターの心拍数が見えること
	Q6. 明るい照明
	Q7. 居心地の悪いベッド
	Q8. 眠れないこと
	Q9. 突然のモニターのアラーム音
	Q10. プライバシーが制限されていること
	Q11. 自分たち以外の辛そうな親が見えること
	Q12. 他の病気の子どもが見えること
	Q13. スタッフの部屋への出入りが激しいこと
	Q14. PICU に入室することを予想していなかったこと
医療的処置	Q15. 子どもにチューブ類が入っていること(例. 栄養チューブ、尿道カテーテル、点滴)
	Q16. 痰を吸引されること
	Q17. 輸液や処置、検査のために子どもに針を刺されること
	Q18. 子どもの胸を押されたり、叩かれたりすること
	Q19. 人工呼吸器で呼吸させられていること
	Q20. 子どもにあざがあること
	Q21. 子どもに傷があること
	Q22. 子どもに切開創があること
スタッフとのコミュニケーション	Q23. 子どもの病気について医師達が十分に話し合っていないように感じる
	Q24. 子どもの状態についてあなたと十分に話していない
	Q25. 子どもの状態について矛盾することを話している
	Q26. 子どもについて何が悪いのかをはっきりと明言しない
	Q27. 子どもの治療についてあなたに十分に話してこない
お子さまの行動と情動の反応	Q28. 子どもが困惑しているように見える
	Q29. 子どもがめそめそと泣いている
	Q30. 子どもが痛がっている
	Q31. 子どもが休めていない
	Q32. 子どもが話すことができない
	Q33. 子どもが泣くことができない
	Q34. 子どもが怖がっているように見える
	Q35. 子どもが悲しそうに見える
	Q36. 子どもが落ち込んでいるように見える
親の役割	Q37. 子どもの世話をしてあげることができない
	Q38. 少しの間でも子どもから離れたくない
	Q39. 子どもを抱きしめることができない
	Q40. 子どもの危険な状態に対して何が最も子どもにとって助けになるのかがわからない
